# Effects of short‐term clomipramine on anxiety‐like behavior, cellular metabolism, and oxidative stress in primary fibroblast cells of male and female rats

**DOI:** 10.14814/phy2.13615

**Published:** 2018-05-10

**Authors:** Ana G. Jimenez, Joshua D. Winward, Dana M. Smith, Christina M. Ragan

**Affiliations:** ^1^ Department of Biology Colgate University Hamilton New York; ^2^ Department of Psychology Neuroscience Program Colgate University Hamilton New York; ^3^ Present address: Psychology Department Purdue University Northwest Westville Indiana

**Keywords:** Anxiety, clomipramine, metabolism, oxidative stress

## Abstract

Anxiety is the most prevalent mental disorder among adults in the United States and females tend to have significantly higher rates of anxiety compared with men. Common treatments for anxiety include usage of selective serotonin reuptake inhibitors (SSRIs) and tricyclic antidepressants, however, sex differences in the efficacy of these drugs exist. In this study, we were interested in determining if acutely manipulating serotonin mechanisms at the whole‐animal level affects cellular metabolism and oxidative stress in primary fibroblast cells from clomipramine‐treated Sprague‐Dawley rats. Our groups included a female and male control group that was injected with a saline solution, a female and male group that was injected with a low dosage of clomipramine, and a female and male group of rats that were injected with a high dosage of clomipramine. We then compared cellular oxygen consumption rates, rates of glycolysis and oxidative stress parameters in primary fibroblasts grown from each of the groups described above. We found that clomipramine‐treated rats had significantly lower rates of glycolysis and glycolytic capacity, regardless of sex. Coupling efficiency was significantly higher in male rats compared with female rats across treatment groups. Our data suggest that in female rats reduced glutathione (GSH) is nonsignificantly reduced, yet lipid peroxidation (LPO) damage still accumulates, meaning that enzymatic antioxidants may be acting to reduce any continual increases in LPO damage. This is a metabolically costly process that may be happening because of our drug treatments. Our results provide further evidence of sex differences in the behavioral and metabolic responses to short‐term clomipramine treatment. Continued investigation into these sex differences may reveal their potential for improving our understanding of how different therapeutic interventions may be better suited for treating males and females.

## Introduction

Anxiety is the most prevalent mental disorder among adults in the United States (Kessler et al. 2005). Furthermore, women have nearly twice the lifetime rate of most anxiety disorders compared with men (Donner and Lowry 2014). Women may also be more vulnerable than men to the adverse effects of stress on anxiety‐related behaviors (Bollini et al. 2017). In addition, women with one anxiety disorder are significantly more likely than men to be diagnosed with another comorbid anxiety disorder (McLean et al. 2011). Common treatments for anxiety include the use of selective serotonin reuptake inhibitors (SSRIs) and tricyclic antidepressants, however, sex differences in the efficacy of these drugs exist. In fact, premenopausal women respond more favorably to SSRIs like sertraline than tricyclic antidepressants such as imipramine, whereas men display the opposite trend (Kornstein et al. 2000; Kokras et al. 2009). In addition to these sex‐specific differences, it is often challenging to use antidepressants because acute treatment may be anxiogenic, low doses may be ineffective, and high doses can increase any adverse effects (Gordon and Hen 2003; Bollini et al. 2017). Cytochrome P450 (CYP450) enzyme variants have been examined in regard to individual variability in response to SSRI treatment. However, there is marginal evidence to suggest links between CYP450 variants and SSRI metabolism, efficacy, and tolerability for patients with depression (reviewed in: Thakur et al. 2007). For this reason, other markers of metabolism may be likely candidates to explain individual variability in responses to SSRI treatment.

Metabolic pathways are highly conserved among animals (Koch and Britton 2008), and any response to phenotypic variability is likely to be a quantitative adjustment rather than a new pathway (Seebacher et al. 2010). Individuals with high trait anxiety demonstrate increases in resting metabolic rate (RMR) compared with low trait anxiety individuals (Schmidt et al. 1996), Mechanistically, this difference may be due to increased activity in the autonomic nervous system in highly anxious individuals and/or increased stimulation of the central nervous system serotonin receptors, which show increased metabolism in the rat (Schmidt et al. 1996). This demonstrates that there may be a specific anxiety phenotype at the whole‐animal level that differs from the cell level phenotype.

Oxidative stress has been implicated as a potential cause of a variety of anxiety phenotypes (Epel 2009; Hovatta et al. 2010; Salim et al. 2010; Salim 2014). As a byproduct of normal oxidative phosphorylation, errant electrons form free‐radicals such as O_2_^−^, OH^−^, or H_2_O_2_ (Harman 2001; Hovatta et al. 2010), can attack DNA, proteins, and lipids. If this damage is not repaired, it may cause impairment of function and ultimately cell death. At low levels, these ROS species are known to be essential for cell survival because of their role in gene regulation, cell signaling, and apoptosis (Dowling and Simmons 2009). However, at high levels, ROS production can potentially overwhelm the antioxidant capacity of the cell and exert oxidative stress changing gene expression and causing structural damage (Dowling and Simmons 2009). Cells inherently contain molecules to combat damage from ROS production, broadly termed the antioxidant system, which include enzymatic antioxidants such as glutathione peroxidase (GPx), superoxide dismutase (SOD), and catalase (CAT) that function by catalyzing the oxidation of less biologically insulting molecules such as H_2_O. Other antioxidant molecules including vitamin E and C act as chain‐breaking antioxidants, which can scavenge for ROS, remove them once they are formed, and further halt propagation of peroxidation (Ersan et al. 2006). Additionally, malondialdehyde (MDA) levels, which positively correlate with lipid peroxidation damage in oxidative stress, are significantly higher in blood drawn from patients with obsessive compulsive disorder (OCD) compared with nonanxious patients (Ersan et al. 2006). Generally, there seems to be a consensus that antioxidant enzymes decrease after chronic stress (Hovatta et al. 2010). Highly anxious mice show a significant increase in ROS production in lymphocytes, granulocytes, and monocytes compared with low‐anxiety mice (Rammal et al. 2008). This increase in also observed in neuronal and glial cells within the brain (Rammal et al. 2008), suggesting that anxiety is also positively correlated with antioxidant enzyme activity. Oxidative stress can also be induced using compounds such as l‐buthionine‐sulfoximine (BSO). Interestingly, systemically injecting BSO also increases anxiety‐like behavior in rats (Salim et al. 2010). Many of these studies suggest that oxidative stress and metabolism can affect the regulation of anxiety (Bouayed et al. 2009).

It has been shown that clomipramine, a tricyclic antidepressant that is selective for serotonin and norepinephrine reuptake, can cause changes to mitochondrial function (Abdel‐Razaq et al. 2011). Thus, in our study, we were interested in determining if acutely manipulating serotonin mechanisms at the whole‐animal level affects cellular metabolism and oxidative stress in primary fibroblast cells from clomipramine‐treated Sprague‐Dawley rats. We also wanted to examine the relationship between anxiety‐related behavior after treatment was completed and cellular metabolism and oxidative stress in primary fibroblast cells in these animals. Our groups included a female and male control group that was injected with a saline vehicle, a female and male group that was injected with a low dose of clomipramine, and a female and male group of rats that were injected with a high dose of clomipramine. We isolated primary fibroblasts for rats included within the above groups and compared oxygen consumption rates, rates of glycolysis and oxidative stress parameters in these cells.

## Materials and Methods

### Animal care

Sprague‐Dawley rats were born and raised at Colgate University and were housed with 1–2 same‐sex cage mates in standard Polypropylene shoe box style cages on site at Colgate University. They all had a large scoop of hardwood chip bedding, eco bedding, a piece of cardboard bent into a tee pee shape for a shelter, and a chewable object such as a nylabone or wood block. Rats were fed Purina 5001 rat chow and water ad libitum. Light cycle was light/dark 12:12 h with lights on at 0700. Humidity was kept at 30% or greater. Room temperature was maintained at 22°C. All protocols were approved by the Institutional Care and Use Committee at Colgate University (Protocol #16‐8).

### Baseline anxiety‐related behavior

Around postnatal day (PND) 95, baseline anxiety‐related behavior was examined on the elevated plus maze between 1200 and 1300 h (Pellow et al. 1985). The elevated plus maze is a 4‐arm maze raised 50 cm above the floor and consists of 2 closed arms with 40 cm‐high walls and 2 open arms. Two opposing arms of the maze are open and lack walls, whereas the other two arms are enclosed by 40 cm high walls. Females’ estrus cycles were monitored by vaginal smear and were tested on a day of diestrus close to PND 95 to reduce the interference of sex hormones on behavior (Marcondes et al. 2001; Tsukamura and Maeda 2001). All animals were tested for 10 min and video recorded using a digital Panasonic SDR‐H100 camera. Following completion of the test, the apparatus was cleaned with 70% ethanol and the subject was returned to its home cage. Behaviors observed were: time spent in open and closed arms and total number of entries to each arm with an entry being recorded if the two front paws entered the arm. Animals that spend a greater amount of time in the closed arms than the open arms were categorized as anxious (Pellow et al. 1985; Costall et al. 1989). Samples sizes included in section below.

### Treatments

To manipulate serotonin (5‐HT) mechanisms globally in these rats, we administered a low or high dose of the tricyclic antidepressant, clomipramine hydrochloride (C7291‐5G, Lot# 089K1221V, Sigma‐Aldrich St. Louis, MO) dissolved in saline or saline control intraperitoneally (IP). Solutions were made fresh daily. Adult rats (approximately ages 95–100 days) were randomly assigned to receive either 1 mg/kg (low dose, Liu et al. 2008) or 15 mg/kg clomipramine (high dose, Kreiss et al. 2015) or 0.9% saline for 7 days via IP injection at around 1200. For all parameters samples sizes were low‐clomipramine females (*N* = 5), low clomipramine males (*N* = 7), high‐clomipramine females (*N* = 5), high‐clomipramine males (*N* = 6), saline females (*N* = 6), and saline males (*N* = 6).

### Post‐treatment anxiety‐related behavior

After 7 days of acute treatment (around PND 100), subjects’ anxiety‐related behavior was examined using a different test, the light‐dark box, to avoid habituation to the elevated plus maze (File 1990; Bourin and Hascoet 2003). Thirty minutes after the final injection between 1200 and 1400 h, subjects’ anxiety‐like behavior was observed and recorded for 10 min. The light‐dark box has one chamber (30 × 34 × 30 cm) made of black Plexiglas with an unattached lid, and one identical size chamber made of white Plexiglas and 717 lx light shining toward the center with no lid. A 10 × 10 cm hole in the wall between chambers allowed the rats to move from one chamber to the other. For each test, the subject was placed in the light chamber facing away from the entrance to the dark chamber. After the test, the apparatus was cleaned with 70% ethanol and the subject was euthanized (see below). Behaviors recorded were: time spent in each chamber, and number of chamber entries with an entry being counted if all four paws entered the chamber. Animals that spend more time in the dark chamber than the light chamber are considered to be anxious.

### Establishment of fibroblast cell lines

Immediately after the light‐dark box test, rats were sacrificed using CO_2_ euthanasia_._ We isolated primary fibroblast cells from skin of rat tails. The samples were placed in cold transfer media (Dulbecco's modified Eagle medium [DMEM], with 4500 mg/L glucose, sodium pyruvate, and 4 mmol/L l‐glutamine supplemented with 10% heat‐inactivated fetal bovine serum, and antibiotics [100 U/mL pen/strep], containing 10 mmol/L HEPES) until processing. To isolate primary fibroblast cells, skin samples were sterilized in 70% ethanol and 20% bleach. Skin was minced and incubated in sterile 0.5% collagenase B overnight in an atmosphere of 37°C 5% O_2_/CO_2_. After incubation, the collagenase mixture was filtered through a sterile mesh (Falcon No. 352340), and centrifuged at 168 g for 5 min. The resulting supernatant was removed, and the pellet was resuspended with 7 mL of mammal media (Dulbecco's modified Eagle medium [DMEM], with 4500 mg/L glucose, sodium pyruvate, and 4 mmol/L l‐glutamine supplemented with 10% heat‐inactivated fetal bovine serum, and antibiotics [100 U/mL pen/strep]). Cells were grown in culture flasks at 37°C in an atmosphere of 5% O_2_/CO_2_. When cells reached 90% confluence, they were trypsinized (0.05%) and cryopreserved at 10^6^ cells/mL in DMEM supplemented with 40% fetal bovine serum and dimethylsulfoxide (DMSO) at a final concentration of 10%. We stored cells in liquid N_2_ prior to assessment of their cellular metabolism and oxidative stress profile (below). All measurements were conducted using cells at passage 2 (P2).

### Metabolic profiles

A Seahorse XF‐96 Extracellular flux analyzer was used to measure the rate of O_2_ consumption and glycolysis in isolated primary dermal fibroblast cells from all rats. Assays were performed prior to experiments to determine the optimal cell seeding density, and optimal concentrations of each compound used. We seeded 20,000 cells per well in duplicate per individual into XF‐96 cell culture plates and allowed cells to attach overnight.

### Extracellular acidification rate

Extracellular acidification rate (ECAR) values were measured in units of mpH, which is the change in pH in the media surrounding the cells due to proton flux in glycolysis. Measurements of ECAR were performed after the cells were equilibrated to running media for 1 h. Running media contained no glucose and 2 mmol/L l‐glutamine in all experiments, pH = 7.4 at 37°C. Baseline rates were measured three times prior to any injections. We first injected a final well concentration of 10 mmol/L glucose into media surrounding cells, which provides a measure of glycolytic rate, and then we injected a final well concentration of 2 *μ*mol/L oligomycin, giving us an estimate of glycolytic capacity in cells. Finally, we injected a final well concentration of 50 mmol/L 2‐DG, a glucose analog that inhibits glycolysis, which provided an estimate of non‐glycolytic acidification (Hill et al. 2012). After measurements were completed, due to differing growth rates of each cell line, we used a Countess II FL cell counter to count the actual final concentration of cells in each well and normalized all rates to a total of 20,000 cells. For each ECAR parameter, we followed the equations supplied by Divakaruni et al. (2014).

### Oxygen consumption rates

Oxygen consumption rates (OCRs) was determined using XF‐96 FluxPaks (37°C) from Seahorse Bioscience. We measured OCRs after cells were equilibrated to running media for 1 h, which contained 10 mmol/L glucose, 1 mmol/L sodium pyruvate, and 2 mmol/L glutamine, pH = 7.4. Baseline measurements of OCRs were made three times prior to injecting a final well concentration of 2 *μ*mmol/L oligomycin, which inhibits ATP synthesis by blocking the proton channel of the Fo portion of the ATP synthase. This analysis can be used to distinguish the percentage of O_2_ consumption devoted to ATP synthesis and the O_2_ consumption required to overcome the natural proton leak across the inner mitochondrial membrane plus any nonmitochondrial O_2_ consumption. Next, we injected a final well concentration of 0.125 *μ*mol/L FCCP, which is an uncoupling agent that disrupts ATP synthesis by essentially collapsing the proton gradient across the mitochondrial membrane leading to uncoupled consumption of energy and O_2_ without generating ATP: this provides a theoretical maximal respiratory rate. Finally, we injected a final well concentration of 0.5 *μ*mol/L Antimycin A, a Complex III inhibitor and rotenone, a Complex I inhibitor. This combination stops mitochondrial respiration and enables nonmitochondrial respiration to be evaluated (Gerencser et al. 2009; Brand and Nicholls 2011; Rogers et al. 2011; Hill et al. 2012).

After measurements were completed, due to differing growth rates of each cell line, we used a Countess II FL cell counter to count the actual final concentration of cells in each well and normalized all rates to a total of 20,000 cells. For each OCR parameter, we followed the equations supplied by Divakaruni et al. (2014). Spare respiratory capacity is the difference between basal respiration and FCCP‐induced maximal respiration and describes the cell's capability to respond to periods of stress or high ATP demand. ATP coupled respiration is the difference between basal respiration and oligomycin sensitive respiration, and describes the ATP demand of the cell (Divakaruni et al. 2014). Coupling efficiency is calculated as the ratio of ATP production and basal respiration. A higher efficiency signifies that respiration is tightly coupled to ATP production. When proton leak is high, coupling efficiency may drop.

### Oxidative stress profiles

For oxidative stress measurements, cells were seeded at 10,000 cells per well and allowed to attach for 24 h prior to any experiments. We used Thermo Scientific™ Nunc™MicroWell™ 96‐Well Optical‐bottom black chimney plates with polymer base for all fluorescent stains. After staining with each fluorescent stain (below) on a separate plate, cells were imaged using a Tecan infinite measure m200 fluorescent plate reader. Due to differing growth rates of each cell line, cells were counted after each experiment using a Countess II FL cell counter and data was normalized to a total of 20,000 cells per well.

### Reduced glutathione

ThiolTracker™ Violet Kit (Glutathione Detection Reagent, Molecular Probes^®^) was used to measure concentration of reduced glutathione (GSH). GSH reduces the oxidized form of the enzyme GPx, which in turn reduces H_2_O_2_. Cells were rinsed with sterile PBS twice and 20 *μ*mol/L ThiolTracker was added to each well. Plates were incubated at 37°C 5% CO_2_/O_2_ for 30 min. Cells were then washed another three times with sterile PBS, and imaged in phenol red‐free FluoroBrite DMEM. Excitation and emission were measured in the violet spectrum at 404/526 nm, respectively (Mandavilli and Janes 2010).

### ROS production

CellROX^®^ Oxidative Stress Reagents kit (Molecular Probes^®^) was used to measure ROS production. CellROX reagents were added directly to the serum‐free medium at a concentration of 5 *μ*mol/L to cells and incubated at 37°C for 30 min. Cells were then washed three times with sterile PBS, and then imaged in phenol red‐free FluoroBrite DMEM. Excitation and emission were in the green spectrum at 488/530 nm, respectively (Gebhard et al. 2013).

### Mitochondrial content

Mitochondrial content was measured using MitoTracker^®^ Mitochondrion‐Selective Probes (Molecular Probes^®^). Cells were stained with a 20 nmol/L of MitoTracker Deep‐Red and incubated at 37°C 5% CO_2_/O_2_ for 60 min. Cells were then washed three times with sterile PBS and imaged in phenol red‐free FluoroBrite DMEM. MitoTracker^®^ excitation and emission were in the red spectrum at 635/670 nm, respectively.

### Lipid peroxidation damage

Lipid peroxidation (LPO) was measured with the Image‐iT^®^ Lipid Peroxidation Kit based on the BODIPY^®^ 581⁄591 reagent. The ratio between red to green indicates the degree of lipid peroxidation. Cells were stained with 10 *μ*mol/L of component A and incubated at 37°C 5% CO_2_/O_2_ for 30 min. Cells were then washed three times with sterile PBS and imaged in phenol red‐free FluoroBrite DMEM. LPO red excitation and emission were 575/610 nm, respectively, and for LPO green was 488/525, respectively (Leiros et al. 2014).

### Statistics

For behavioral comparisons between sexes, *t*‐tests were performed. 2X2 ANOVAs were then used to analyze sex X treatment effects. Data from every assay were first tested for normality using a Kolmogorov‐Smirnov test. If not normal, the data were log‐transformed prior to other statistical analyses to meet assumptions of an ANCOVA. For this study, we log‐transformed basal respiration, proton leak, ATP production, coupling efficiency, glycolysis, glycolytic capacity, nonglycolytic acidification, reduced glutathione, and LPO damage data sets prior analyses. These data were then analyzed using a two‐way ANCOVAs by sex and treatment, with body mass and age at collection as co‐variates. Results were considered significant if *P* < 0.05. We performed statistical tests using SPSS 24.0.

## Results

### Baseline anxiety‐related behavior

Males and females did not differ in the average number of entries to the closed arms (*t*(32) = 0.84, *P* > 0.05;) nor the open arms (*t*(32) = 0.97, *P* > 0.05) of the elevated plus maze. In addition, there were no sex differences in the relative time spent in the open arms (*t*(33) = 0.11, *P* > 0.05).

### Post‐treatment anxiety‐related behavior

**Figure 1:**
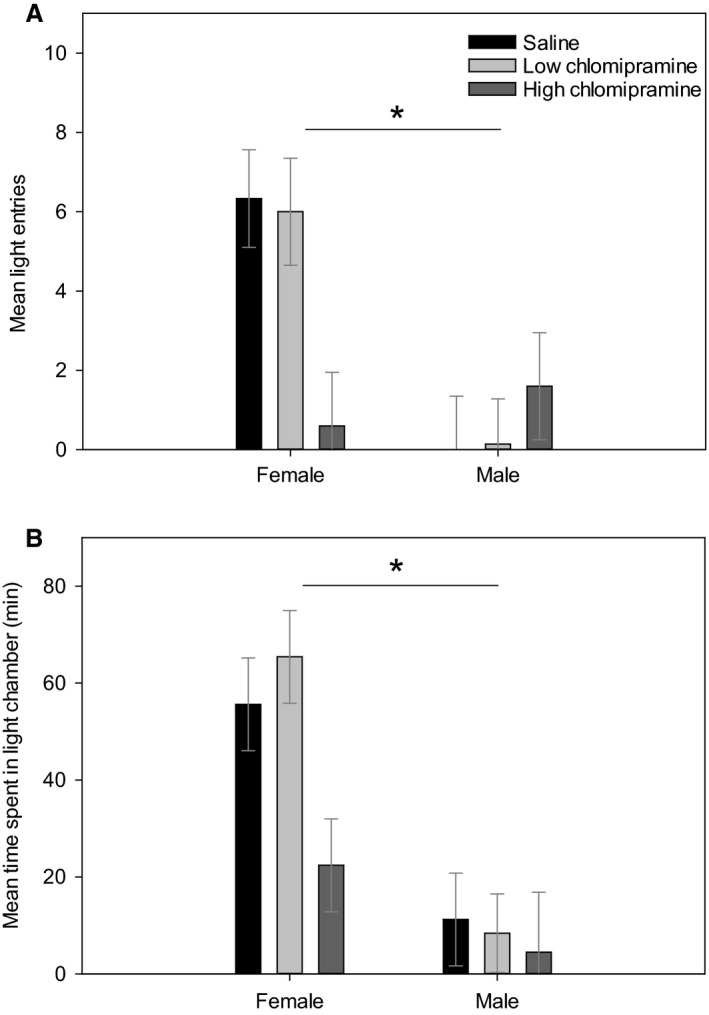
Panel (A) Shows light chamber entries in the light‐dark box for males and females after 7‐day treatment with either saline or clomipramine. Panel (B) Shows time spent in the light chamber in males and females after 7‐day treatment with either saline or clomipramine. For all parameters samples sizes were low‐clomipramine females (*N* = 5), low‐clomipramine males (*N* = 7), high‐clomipramine females (*N* = 5), high‐clomipramine males (*N* = 6), saline females (*N* = 5) and saline males (*N* = 6). Values are presented as averages ± SEM. Asterisk (*) highlight significant differences.

There was a significant sex X treatment interaction for light chamber entries (*F*(2, 27) = 4.82, *P* < 0.02; Fig. 1A). Short‐term saline treated males made significantly fewer entries into the light chamber than did short‐term saline females (*t*(9) = 2.64, *P* < 0.03; Fig. 1A). In addition, males treated with a low dose of clomipramine made significantly fewer entries into the light chamber than did females given the same treatment (*t*(12) = 3.99, *P* < 0.003 Fig. 1A). There was also a significant sex X treatment interaction indicating that low‐dose clomipramine treated males spent less time in the light chamber than did low‐dose clomipramine treated females (*t*(10) = 6.44, *P* < 0.00007; Fig. 1B).

### Body mass

Female rats in the low‐clomipramine treatment group weighed 255.4 ± 8 g, whereas males in the same treatment group weighed 384.7 ± 4.9 g. Female rats in the high‐clomipramine treatment group weighed 250 ± 6.9 g, whereas males in the same treatment group weighed 396.8 ± 16.1 g. Saline control females weighed 247.8 ± 9.7 g, whereas saline control males weighted 385.8 ± 8.4 g.

### Age at collection

Female rats in the low‐clomipramine group were collected at 98.6 ± 0.75 days of age, whereas male rats in the same treatment group were collected at 108.7 ± 3.8 days of age. Female rats in the high‐clomipramine treatment group were collected at 107.4 ± 5.2 days of age, whereas males in the same treatment group were collected at 106.3 ± 4.2 days of age. Saline control females were collected at 105.6 ± 4.6 days of age, whereas saline control males were collected at 105.8 ± 4.3 days of age.

### Extracellular acidification rate

We found no significant differences between sexes in glycolysis (*F* = 0.021, *P* = 0.885), glycolytic capacity (*F* = 0, *P* = 0.991), and nonglycolytic acidification (*F* = 0.436, *P* = 0.515).

**Figure 2:**
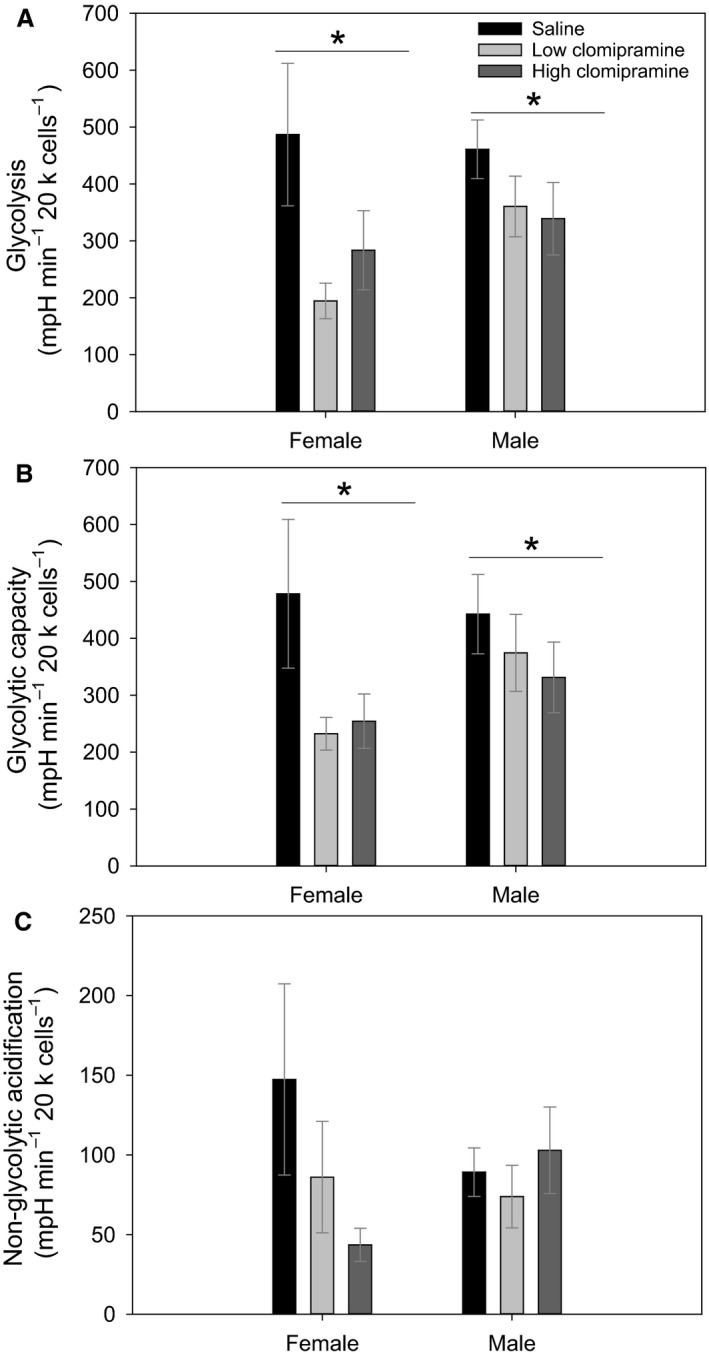
Panel (A) shows glycolysis rates after the injection with glucose. Panel (B) shows potential glycolytic capacity after the injection with Oligomycin, and Panel (C) shows nonglycolytic acidification after the injection of 2‐DG. For all parameters samples sizes were low‐clomipramine females (*N* = 5), low‐clomipramine males (*N* = 7), high‐clomipramine females (*N* = 5), high‐clomipramine males (*N* = 6), saline females (*N* = 6) and saline males (*N* = 6). Values are presented as averages ± SEM. Asterisk (*) highlight significant differences.

We did find significant differences across treatments in glycolysis (*F* = 5.487, *P* = 0.010, Fig. 2A), glycolytic capacity (*F* = 3.402, *P* = 0.048, Fig. 2B) with saline‐treated rats exhibiting higher glycolysis and glycolytic capacity versus clomipramine‐treated animals but there were no significant differences in nonglycolytic acidification among treatment groups (*F* = 1.435, *P* = 0.256 Fig. 2C).

### Oxygen consumption rates

**Figure 3:**
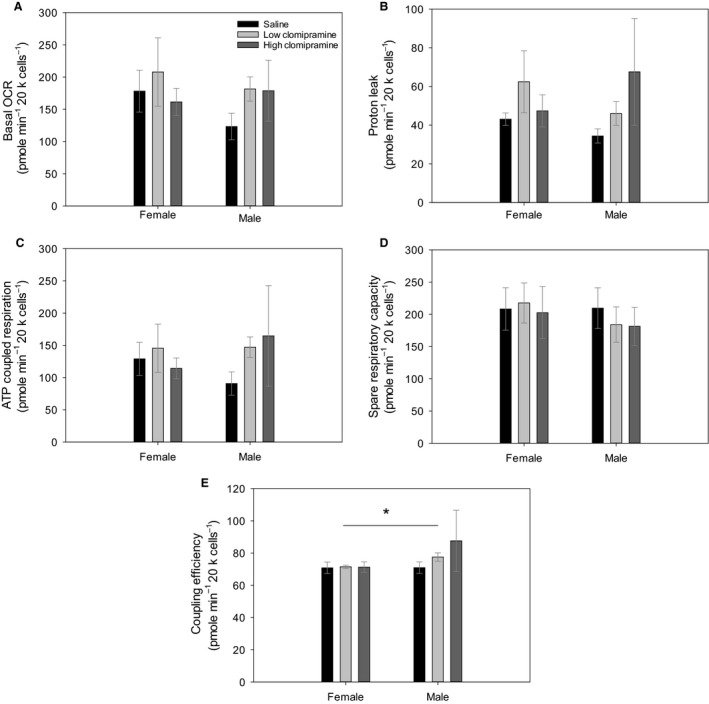
Panel (A) shows basal oxygen consumption rates (OCR) for each of our treatments across sex differences. Panel (B) shows measurements of proton leak after an injection with Oligomycin. Panel (C) shows ATP coupled respiration as the difference between basal respiration, and oligomycin‐sensitive respiration. Panel (D) shows spare respiratory capacity as the difference between basal respiration and FCCP‐induced maximal respiration and Panel (D) shows coupling efficiency calculated as the ratio of ATP production and basal respiration. For all parameters samples sizes were low‐clomipramine females (*N* = 5), low‐clomipramine males (*N* = 7), high‐clomipramine females (*N* = 5), high‐clomipramine males (*N* = 6), saline females (*N* = 6) and saline males (*N* = 6). Values are presented as averages ± SEM. Asterisk (*) highlight significant differences.

We found no significant differences between sexes in basal respiration (*F* = 0.018, *P* = 0.895, Fig. 3A), proton leak (*F* = 0.372, *P* = 0.547 Fig. 3B), ATP coupled respiration (*F* = 1.323, *P* = 0.260 Fig. 3C), and spare respiratory capacity (*F* = 0.775, *P* = 0.386, Fig. 3D). However, we did find sex differences between coupling efficiency with males showing higher coupling efficiency compared to females (*F* = 19.24, *P* < 0.0001, Fig. 3E).

We found no significant differences across treatments in basal respiration (*F* = 1.180, *P* = 0.323), proton leak (*F* = 0.753, *P* = 0.481), ATP production (*F* = 1.704, *P* = 0.201), spare respiratory capacity (*F* = 0.404, *P* = 0.672) and coupling efficiency (*F* = 0.985, *P* = 0.386).

### Oxidative stress profiles

**Figure 4:**
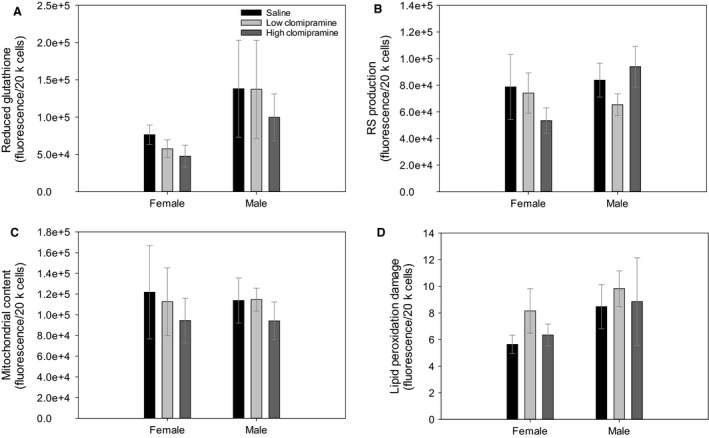
Panel (A) shows concentration of reduced glutathione (GSH). Panel (B) shows basal ROS production. Panel (C) shows mitochondrial content in primary fibroblasts and Panel (D) shows lipid peroxidation damage. For all parameters samples sizes were low‐clomipramine females (*N* = 5), low‐clomipramine males (*N* = 7), high‐clomipramine females (*N* = 5), high‐clomipramine males (*N* = 6), saline females (*N* = 6) and saline males (*N* = 6). Values are presented as averages ± SEM.

We found no significant differences between sexes in reduced glutathione (*F* = 3.533, *P* = 0.071, Fig. 4A), ROS production (*F* = 0.228, *P* = 0.637 Fig. 4B), mitochondrial content (*F* = 0.425, *P* = 0.520 Fig. 4C), and LPO damage (*F* = 0.815, *P* = 0.375 Fig. 4D).

We found no significant differences across treatments in GSH (*F* = 0.389, *P* = 0.682), ROS production (*F* = 0.411, *P* = 0.667), mitochondrial content (*F* = 0.397, *P* = 0.676), and LPO damage (*F* = 0.917, *P* = 0.412). Age at collection explained 29.1% of the variation around LPO damage.

## Discussion

In this study, we looked at the effects of whole‐animal week‐long clomipramine treatment on cellular metabolism and oxidative stress in primary fibroblasts. Although sex differences in anxiety and treatment response are known, they have not been extensively studied. Women and nonhuman female mammals are underrepresented in biomedical research due to the assumption that findings in males apply to females (Beery and Zucker 2011). Since women suffer from anxiety disorders more often and they receive antidepressants more frequently than do men (Bollini et al. 2017), it is critical to understand the ways in which these treatments affect both males and females. Importantly, the National Institutes of Health (NIH) have encouraged the use of both sexes in research (Clayton and Collins 2014), especially as female subjects (rodent and primate) are often excluded from studies, yet can have very different behavior and physiology compared to males. Our study is novel for two reasons: first, we incorporate female rats into our study and secondly, we look at primary fibroblasts as a model cell type to examine the effects of antidepressant treatments in vivo after assessing behavioral whole‐animal responses to the drug. We found that (1) glycolysis and glycolytic capacity significantly decreases with clomipramine treatments, (2) coupling efficiency is significantly different between male and female rats, and (3) reduced glutathione decreases in female rats compared with male rats, though not significantly.

Saline and low‐dose clomipramine‐treated males made significantly fewer entries into the light chamber and spent less time in the light chamber compared to their female counterparts. Whereas we expected that males would have been more exploratory and less anxious, the limited number of entries into the light did not support this prediction. Taken together, the pattern of entry behavior has two possible explanations: The first possibility is that females displayed hyperlocomotion. Studies have shown that anxiety is strongly associated with hyperlocomotion therefore, many entries into the light may actually indicate anxiety‐like behavior in the female rats (Joshi et al. 2005; Ishikawa et al. 2014).

An alternative explanation for our behavioral results is that clomipramine has detrimental effects on the locomotion of males. Human studies have shown that tricyclic antidepressants that are selective for both norepinephrine and serotonin uptake, including clomipramine, are more effective for relief of symptoms than are SSRIs, but that they are tolerated more poorly (Anderson 1998). Likewise, SSRIs can dose‐dependently enhance locomotor activity, whereas clomipramine only slightly increases locomotion (Brocco et al. 2002). Elevated locomotion was only seen at a 10 mg/kg dose of clomipramine (we used 5 mg/kg and 15 mg/kg), while any other doses were inactive similar to other tricyclics that were assessed (Brocco et al. 2002). Therefore, males may display attenuated locomotion as an adverse consequence of clomipramine treatment while other behaviors are improved with treatment. This interpretation contrasts with our initial prediction than males would respond favorably than females to clomipramine because it is a tricyclic antidepressant. However, this differential responsivity and tolerability of antidepressant classes was found in a human population with chronic major depression or double depression (Kornstein et al. 2000) so it is possible that this sexual dimorphism is not present in subclinical rats.

High‐anxiety mice show a decrease in the expression of seven glycolytic enzymes compared with low‐anxiety rats (Filiou et al. 2011). We also see a decrease in glycolytic pathways in clomipramine‐treated rats compared with saline controls, though we do not see a concomitant upregulation of oxygen consumption pathways as Filiou et al. (2011) saw in mice synaptosomes. However, other studies have demonstrated that high energy phosphate compounds are not altered in the brain following treatments with clomipramine (Maekawa et al. 1980), thus, we may not expect to see an upregulation of oxidative pathways in primary fibroblast cells, if brain cells did not upregulate high energy compounds after treatment with the same antidepressant. Clomipramine at concentrations higher than 5 mg/kg has been associated with significant increases in blood glucose levels compared with saline controls in mice immediately after injection (Sugimoto et al. 2003), thus, the decrease in glycolysis that we show in our clomipramine‐treated rats may suggest that after a week of treatment with clomipramine, rats adapt to any extra glucose induced by treatment with the antidepressant and are even able to downregulate the glycolytic metabolism after 7 days of treatment. This is significant when considering that there is often a delay between the onset of treatment with antidepressant drugs and a behavioral reaction to the drug (Berton and Nestler 2006).

Cellular energy can come from glycolysis, the citric acid cycle, and oxidative phosphorylation (Schmidt et al. 1996). It has been shown that clomipramine causes changes in mitochondrial function (Eto et al. 1985; Abdel‐Razaq et al. 2011). In isolated mitochondria from rat hearts, mitochondrial complex I was the most sensitive to antidepressant inhibition, whereas complex II and III showed decreased activity with antidepressants, and complex IV was inhibited with antidepressant drugs at higher concentrations (Abdel‐Razaq et al. 2011) suggesting that most of the ROS production complexed in the mitochondria are affected by antidepressant drugs. However, we make use of tissue culture methods for two reasons. First, a nucleus from a cell isolated from skin of any species contains a nucleus with the animals’ genotype, which contains a genome of an evolutionary endpoint in the environment that the animal has animal experienced, including any in vivo treatments. Secondly, while respiration of isolated mitochondria can be measured, as shown by the studies above, we opted to examine the metabolic phenotypes in the whole cell as all of the regulatory elements are still intact. Thus, our experiments demonstrate the actual effects of clomipramine after treatment of the whole‐animal. We found that primary fibroblasts from male rats had a significantly higher coupling efficiency compared with cells from female rats, meaning that male rats have tightly coupled respiration and ATP production. These sex differences in coupling may be related to the differences in anxiety‐like behavior observed in the male versus female rats after completion of treatment with low doses of clomipramine. Interestingly, although the both male clomipramine groups experience greater coupling efficiency compared with females treated with clomipramine, the low‐dose clomipramine‐treated males exhibited higher anxiety‐like behavior than their female counterparts. Subunits of all electron transport chain complexes and the ATP synthase have shown higher expression in high‐anxiety mice compared with low‐anxiety mice (Filiou et al. 2011), thus, mitochondria may play a key role in altering anxiety‐related behavior.

ROS form due to the electronic structure of O_2_, and they form into superoxide, hydrogen peroxide and/or hydroxyl radical. All of these molecules are capable of causing cellular damage to adjacent macromolecules if not properly eradicated (Halliwell and Gutteridge 2007). In lipid molecules, H_2_O_2_ clips COO^−^ groups off fatty acids and lipid radicals can set off a chain‐reaction and propagate damage to other molecules such as DNA and proteins (Halliwell and Gutteridge 2007). GSH is able to ameliorate H_2_O_2_ into H_2_O so as to prevent the propagation of lipid peroxidation damage. Our data suggests that in female rats GSH is reduced, yet LPO damage still accumulates, meaning that enzymatic antioxidants may be acting to reduce any continual increased in LPO damage. This is a metabolically costly process that may be happening as a result of our drug treatments. At the whole‐animal level, oxidative stress has been induced in rats by xanthine and/or BSO supplementation and intraperitoneal injections. After a week of these treatments, rats had increased blood pressure, insulin resistance, and corticosterone levels, indicating that increases in oxidative stress led to an anxious phenotype (Salim et al. 2010). Highly anxious mice have decreased total antioxidant capacity in plasma compared with low‐anxiety mice (Filiou et al. 2014). However, other studies have not seen an effect on oxidative stress parameters in patients treated with several serotonin‐acting antidepressants including venlafaxine, reboxetine, or sertraline (Sarandol et al. 2007). These authors concluded that their 6‐week treatment with antidepressants may not have been long enough to see effects on oxidative stress in their patients (Sarandol et al. 2007).

Our treatment paradigm introduced the confound of injection stress. Recent investigations have found that there are individual differences in affective response to chronic injection stress. In a recent study, affective response was assessed by the locomotor response to the moderate stress of a one‐hour novel environment exposure. Following 14 days of I.P. injections of 0.9% saline, high locomotion responder rats showed an increase in depressive‐like behavior during a forced swim test, whereas low locomotion responders displayed a decrease in depressive‐like behavior (Aydin et al. 2015). Although we were unable to control for injection stress, we did not observe increased anxiety‐like behavior following longer periods of injections (i.e., chronic treatment for 21 days in a separate cohort; data not shown) suggesting that injection stress does not explain the anxiety differences found in our study. Another limitation is that we relied on natural variability in anxiety among randomly‐selected rats and did not induce or manipulate anxiety or depression. As a result, it may be more difficult to capture more dramatic anxiogenic, anxiolytic, and metabolic effects of clomipramine.

Overall, our results provide further evidence of sex differences in the behavioral and metabolic responses to short‐term clomipramine treatment. Continued investigation into these sex differences may reveal their potential for improving our understanding of how different therapeutic interventions may be better suited for treating males and females.
